# Variables Involved in the Discordance between HbA1c and Fructosamine: The Glycation Gap Revisited

**DOI:** 10.1371/journal.pone.0066696

**Published:** 2013-06-12

**Authors:** Carles Zafon, Andreea Ciudin, Silvia Valladares, Jordi Mesa, Rafael Simó

**Affiliations:** 1 Diabetes and Metabolism Research Unit (VHIR) and Department of Endocrinology. Hospital Vall d’Hebron and Universitat Autònoma de Barcelona, Barcelona, Spain; 2 Centro de Investigación Biomédica en Red de Diabetes y Enfermedades Metabólicas Asociadas (CIBERDEM), ISCIII, Spain; University of KwaZulu-Natal, South Africa

## Abstract

**Aims:**

Glycation gap (GG) is defined as the difference between the measured level of HbA1c and the level that would be predicted from its regression on the fructosamine level. The aims of the study were: 1) To determine the reproducibility and consistency of GC; 2) To discover factors related to GG value. Given that metformin might increase glucose transport through the erythrocyte membrane, this treatment was also considered in the analyses of the results.

**Methods:**

GG was calculated in two blood samples separated 30.6 (SD 7.3) weeks, obtained in 508 type 2 diabetic patients. The following variables were considered: HbA1c, fructosamine, glucose, creatinine, hematological parameters and treatment with metformin. Multivariate and logistic regression analyses were performed to explore the variables independently related to CG.

**Results:**

GG was reproducible and consistent over time. Creatinine, mean corpuscular hemoglobin concentration (MCHC), and glycemia (inverse relationship); and HbA1c and treatment with metformin (direct relationship) were independently related to GG. Patients treated with metformin showed higher HbA1c values, despite similar fructosamine concentrations, than patients not treated with the drug.

**Conclusions:**

GG is independently related to serum levels of creatinine, MCHC and treatment with metformin. The spurious effect of metformin on Hb glycation could have serious clinical implications and should be considered when interpreting the results of clinical trials.

## Introduction

Hemoglobin (Hb)A1c is adult Hb (HbA) with glucose bound to its β chain N-terminal valine, resulting from non-enzymatic glycation in erythrocytes [1]. Hence, HbA1c concentration reflects the concentration of glucose to which erythrocytes are exposed over their lifespan. More than 30 years ago, HbA1c measurement was introduced into clinical practice as a marker of glycemic control. Since then, HbA1c has been the cornerstone of the management of diabetes mellitus. It is accepted that HbA1c correlates with average blood glucose levels over the preceding 2–3 months. Furthermore, HbA1c is related to the risk of microvascular (in both type 1 and type 2 diabetes) and macrovascular (at least in type 1 diabetes) complications [2], [3]. More recently, HbA1c determination has been proposed as a criterion for diabetes diagnosis [4]. However, the correlation between glycemia and HbA1c is not perfect [5]. It has been reported that numerous factors influence HbA1c concentration. For instance, in both diabetic and non-diabetic subjects, HbA1c levels are genetically determined, with heritability >50% [6]. Other factors such as age, hemoglobinopathies, drugs, and some diseases may affect HbA1c. Moreover, HbA glycation in erythrocytes depends on glucose transport into erythrocytes and on intracellular glucose and protein metabolism. In this regard, a recent report have shown that hemoglobin glycation may partly explain the discordance between HbA1c measurements and oral glucose tolerance test when used for dysglycaemia diagnosis [7].

Many proteins, other than HbA, also suffer a non-enzymatic glycation process. Fructosamine is a ketoamine formed when the carbonyl group of glucose reacts with an amino group of a protein, thus forming glycated serum proteins (mainly albumin) [8]. Fructosamine determination is the most widely used alternative to HbA1c. Some studies have attempted to interpret the parallel measurement of fructosamine and HbA1c. Fructosamine correlates rather well with HbA1c, and Pearson correlation coefficient (*r*) ranges from 0.55 to 0.88 [9]–[12]. The absence of total correlation has been attributed to the different period over which HbA1c and fructosamine average glycemia. However, it has been postulated that differences may also respond to other factors such as individual variations between intracellular and extracellular protein glycation capacity [13]. Fructosamine measures extracellular glycated protein and, therefore, its plasma levels are not related to extracellular–intracellular glucose dynamics.

Cohen et al [14] defined the term glycation gap (GG; formerly glycosylation gap) as the difference between the measured HbA1c and the HbA1c predicted from the measure of fructosamine, based on the HbA1c–fructosamine regression equation. GG could reflect the variance in HbA1c that occurs in processes in which both extracellular and intracellular compartments are involved in comparison with those limited to the extracellular compartment. A substantial part of GG is unrelated to heritable causes and has no relationship with HbA1c [15]–[17]. Therefore, GG could be useful for identifying the factors accounting for the discrepancy between fructosamine and HbA1c levels.

On these bases, the aims of the present study were: 1) To determine the reproducibility and consistency of GG in type 2 diabetic patients; 2) To identify analytical parameters apart from HbA1c, related to GG value. 3) Given that metformin could facilitate glucose transport through the erythrocyte membrane, we also wanted to examine the impact of metformin treatment on GG.

## Materials and Methods

### Patients

Patient’s data were obtained and handled according to guidelines of the Human Ethics Committee of our Hospital. The Human Ethics Committee of our Hospital (Comitè ètic d'Investigació Clínica, CEIC, Hospital Universitari Vall d'Hebron) waived the need for written informed consent and ethics approval, because information obtained in routine analyses was recorded by the investigator in such a manner that subjects cannot be identified, directly or through identifiers linked to the subjects.

A total of 508 consecutive type 2 diabetic patients attending our outpatient clinic from April to June 2011 were included in the study. In all patients two laboratory analyses including HbA1c and fructosamine were performed within 5–7 months after study entry. Exclusion criteria only included known hemoglobinopathies, anemia (Hemoglobin <11.8 g/dL), hypoproteinemia or hypoalbuminemia (total proteins or albumin lower than 6.6 g/dL or 3.4 g/dL, respectively) and patients without completed laboratory data. Clinical data and treatments received were obtained from electronic health records.

### Laboratory analyses

HbA1c, fructosamine, glucose, creatinine, and hematological parameters such as hematocrit, hemoglobin, red distribution width (RDW), and mean corpuscular hemoglobin concentration (MCHC) were measured simultaneously in two separate blood samples (D1 and D2) during routine clinical care. Biochemical parameters were measured using an Olympus AU5400 automatic biochemistry analyzer Olympus corp., Tokyo, Japan), and hematological parameters were evaluated on an LH-750 Beckman Coulter hematology analyzer (Beckman Coulter, USA). HbA1c was measured by HPLC using and ADAMS-A1c (A8180V; Menarini Diagnostics, Italy). Fructosamine was measured by a colorimetric method (Olympus AU5400). In each determination, GG was calculated by the method proposed by Cohen et al [14].

### Statistical analysis

Demographics and disease characteristics were summarized using mean (SD) for continuous and proportions for categorical variables. Continuous variables included basal glucose, HbA1c, D1-D2 HbA1c difference, fructosamine, creatinine, MCHC, RDW, age, and diabetes duration. Categorical variables included sex and type of treatment: insulin, metformin, sulphonylureas, and other oral hypoglycemic agents (OHAs). The GG was evaluated as a continuous variable (the difference between measured HbA1c and HbA1c predicted from the fructosamine level based on a regression equation), but also as a categorical variable within the following three ranges: < –0.5 (negative), –0.5 to 0.5 (neutral), and >0.5 (positive). Reproducibility of the GG was assessed through the correlation of this factor between D1 and D2. Consistency in the GG (relation of GG as a categorical variable between both blood samples) was assessed with a correlation model, the GG in D1 (on the x axes) plotted against the product of both GGs (on the y axes). Thus, the product of any two concordant GGs (either positive or negative) would always be positive; whereas it would be negative only in discordant cases (GG positive in one determination but negative in the other).

Factors related to GG value were determined with a bivariate analysis. The χ^2^ test was performed for categorical variable analysis. The Student’s *t* test and ANOVA, and linear regression techniques were used when the explanatory variables were quantitative. Predictors that achieved a p value <0.05 in bivariate analysis were assessed for inclusion in the multivariate model. The multivariate linear model was constructed using a stepwise regression technique. Moreover, for GG as a categorical variable (positive, neutral and negative) a multinomial logistic regression model was constructed. In regression techniques, the coefficient of determination or square of the multiple correlation coefficient (*R* ^2^) was calculated.

## Results

Table 1 summarizes the baseline characteristics of the 508 type 2 diabetes mellitus included in the study. Data on HbA1c, fructosamine, and the remaining laboratory parameters for the two paired samples are shown in Table 2. Both analytical determinations were separated by a mean of 30.6 (7.3) weeks.

**Table 1:** Baseline characteristics of patients with type 2 diabetes mellitus included in the study.

n	508
Gender	
Women (%)	226 (44.5)
Men (%)	282 (55.5)
Age (years)*	66.1 (11.3)
Duration of diabetes (years)*	13.6 (9.2)
Treatment	
Insulin (%)	344 (67.7)
Metformin (%)	306 (60.2)
Other oral antidiabetic agents (%)	202 (39.8)

* Median (SD).

**Table 2:** HbA1c, fructosamine, and the remaining laboratory parameters for the two paired samples (first and second).

n = 508	First	Second
Glycaemia	8.5 (2.9)	8.5 (2.8)
HbA1c	7.50 (1.29)	7.50 (1.23)
Fructosamine	291.82 (53)	283.4 (53)
Hemoglobin	134 (17)	133 (15)
Hematocrit	39.50 (4.6)	39.20 (4.4)
MCHC	340 (7)	340 (7)
DVH	14.40 (1.6)	14.50 (1.6)
Creatinine	92.8 (44)	91.9 (53)
HbA1c-Fructosamine regression equation	HbA1c = 2.40+0.0175 F	HbA1c = 2.56+0.0174 F
	r = 0.71; *p* = 0.00	r = 0.75; *p* = 0.00
FHbA1c	7.50 (0.9)	7.49 (0.9)
Glycation Gap	– 0.003 (0.9)	0.01 (0.8)

### Reproducibility and consistency of GG

**Figure 1:**
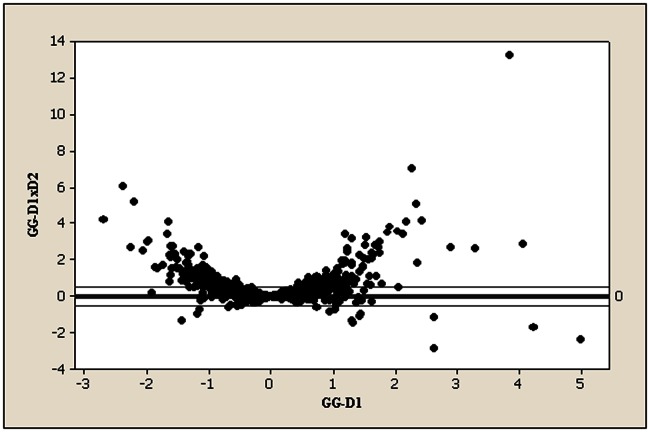
Consistency in the glycation gap (GG) between the two consecutive determinations. The GG of the first sample on the x axis (GG-D1), against the product of both GGs on the y axis (GG-D1×D2).

HbA1c and fructosamine were moderately correlated in D1 and D2 (*R* ^2^ = 0.49 and 0.44, respectively). Regression equations between both parameters are shown in Table 2. Accordingly, the resultant GG was –0.003 (0.9) in D1 and 0.01 (0.8) in D2 (paired Student’s *t* test, p = 0.58). To assess the reproducibility of glucose control parameters, we estimated the correlation between those parameters in the two blood samples. Thus, *r* was 0.45, 0.56, 0.63 and 0.65 for basal glycemia, HbA1c, fructosamine and the GG, respectively. It is interesting to note that the highest correlation coefficient was found for the GG. Moreover, to examine consistency in the GG a specific correlation model was used (Figure 1). Discordance in the direction of the GG between the two determinations is annotated as a negative figure on the y axis (product of the two GGs). A negative value of the product >0.5 occurred in only 11 patients (2%).

### Parameters influencing the GG

In the bivariate analysis, basal glucose (p = 0.001), creatinine (p = 0.008), MCHC (p = 0.001), RDW (p = 0.008), HbA1c difference (p = 0.001), age (p = 0.001), time of diabetes evolution (p = 0.003), gender (p = 0.004), and treatment with metformin (p = 0.001) correlated significantly with the GG. In the multivariate logistic regression analysis, basal glucose (p = 0.001), creatinine (p = 0.003), MCHC (p = 0.002), D1-D2 HbA1c difference (p = 0.001), sex (p = 0.017), and treatment with metformin (p = 0.022) retained significance. When HbA1c was added to the multivariate model, only HbA1c (p = 0.001), MCHC (p = 0.001), basal glucose (p = 0.006), creatinine (p = 0.000) and metformin treatment (p = 0.001) were significant. The results of this multivariate analysis are displayed in Table 3. *R* ^2^ for this model was 0.52 (*R* ^2^ for HbA1c alone was 0.44). In the logistic regression model (the GG categorized into positive, neutral and negative), four of the five factors were statistically significant for positive GG: HbA1c (OR 0.16; 95% CI 0.12–0.22), MCHC (OR 1.87; 95% CI 1.44–2.41), creatinine (OR 1.94; 95% CI 1.33–2.84), and metformin treatment (OR 0.48; 95% CI 0.32-0.73). Basal glucose was also included in the model and was not a statistically significant factor. The results showed that the GG was inversely related to creatinine and MCHC but was directly related to HbA1c and treatment with metformin.

**Table 3:** Multivariate analysis for parameters influencing the glycation gap (GG).

Variable	Coefficient	95% CI	*P* value
Intercept	1.913	–0.251; 4.078	0.083
MCHC	–0.151	–0.213; –0.089	0.000
Glucaemia	–0.001	–0.002; –0.000	0.006
Creatinine	–0.218	–0.299; –0.138	0.000
Treatment with metformin	0.215	0.110; 0.319	0.000
HbA1c	0.48129	0.431; 0.530	0.000

r for the model  = 0.72; R^2^ for model  = 0.52.

We found that metformin was related to an increase in the GG. As a measure of HbA1c adjusted for fructosamine, patients treated with metformin showed higher HbA1c values, despite similar fructosamine concentrations, than patients not treated with the drug. Moreover, the fructosamine–HbA1c quotient was significantly lower in patients treated with metformin, and this effect was not present in the group of sulphonylureas or other OHAs (data not shown).

## Discussion

In the present study we provide evidence that apart from glucose and Hb A1c, MCHC and serum creatinine levels are variables independently related to the GG. In addition, we found that metformin is also an independent variable which influences the GG directly. Therefore, for similar fructosamine concentrations, those patients treated with metformin showed higher HbA1c values than patients receiving other glucose-lowering treatments. Finally, we have shown that the GG is consistent and reproducible over time (∼6 months), thus extending and reinforcing recent reports on this issue [15]–[17] and pointing to the persistence of the underlying mechanisms.

GG is computed as the residual difference between the observed level of A1c and that predicted from its regression with fructosamine. It has been argued that the GG must be strongly correlated with A1c and could be considered only as a surrogate for A1c [18]. However, Chalew et al [19] have found a correlation of 0.7 between HbA1c and the GG. Thus, the coefficient of determination (*R* ^2^) was 0.49. In the study of Rodríguez-Segade et al [16] *R* ^2^ was 0.59 and Cohen et al reported the lowest correlation between both variables in non-diabetic twins (*r* = 0.48; *R* ^2^ = 0.23) [20]. In the present study *R* ^2^ was 0.44. *R* ^2^ indicates how much of the variation in GG is explained by HbA1c. Accordingly, HbA1c explains approximately half of the GG variation and other factors must explain the other ∼50%. This is not surprising because glycation of Hb is more complex than glycation of circulating proteins. There are three well-defined conditions that can alter final HbA1c levels: the amount of glucose entering the erythrocytes, the rate of glycation-deglycation inside the cell, and average of erythrocyte lifespan [1]. Notably, it has recently been reported that interindividual heterogeneity in glucose gradients across RBC membranes may affect hemoglobin glycation [21]. Our results indicate that besides the parameters of glucose control, creatinine, MCHC, and metformin treatment influence HbA1c levels.

Renal failure may affect the accuracy of the HbA1c values because of several factors, such as anemia, assay interference from uremia, uremia-induced hemoglobin modification, and hemodialysis [22]. However, the most influential variable is shortened blood cell survival [23]; a situation that influences the HbA1c–fructosamine correlation. Shima et al [24] have recently found a lower value of HbA1c relative to glycemic control in diabetic patients with end-stage renal disease in comparison with diabetic patients without renal dysfunction. In the present study, we found that creatinine levels were independently and inversely related to the GG in type 2 diabetic patients with creatinine ranging from 38 to 487 µmol/L, mean 92(44), thus suggesting that GG is already altered at early stages of renal impairment, which is in agreement with a recent report by Nayak et al [15]. In fact, an increase of 44 µmol/L in creatinine levels accounts for a reduction of 0.128 in GG. These findings could have significant clinical implications because it would mean that HbA1c of diabetic patients with renal failure could be underestimated. In order to confirm this statement, a specific study addressed to investigating the impact of renal function on GG by using more accurate measurements of renal function such as glomerular filtrate seems warranted.

Qualitative and quantitative variations in Hb might affect HbA1c levels. In this regard, anemia due to iron deficiency tends to increase HbA1c [25]–[27], whereas other forms of anemia are associated with lowering HbA1c [28]. We have evaluated two hematological parameters, the RDW and the MCHC. The RDW detects the heterogeneity of red cell size and we found that this parameter was related to GG in bivariate analysis but not in the multivariate model. The MCHC reflects the average weight of hemoglobin per unit volume of erythrocytes and this was an independent variable in multivariate models. The MCHC was inversely correlated with the GG, thus suggesting that the higher the MCHC, the lower the glycation of Hb. In our sample, every decrease of 10 g/dL in MCHC explains an increase of 0.157 in the GG. This finding indicates that the MCHC should be taken into account when interpreting HbA1c levels in type 2 diabetic patients.

Metformin is currently used in the first-line treatment of type 2 diabetes. Although the reduction in HbA1c achieved by using metformin is very similar to other oral glucose-lowering agents (∼1%) [29], patients under treatment with metformin present a lower diabetes-related endpoint risk and cardiovascular mortality risk [30]. This characteristic has been attributed to a pleiotropic effect that includes a reduction in blood pressure, an improvement in lipid profile, and a reduction in the low-grade inflammation state (by decreasing proinflammatory cytokines and benefiting endothelial function) [31]. However, our results suggest that metformin facilitates Hb glycation and, therefore, patients under treatment with metformin might actually have better glycemic control than that revealed by HbA1c. In this regard, there is experimental evidence showing that metfomin improves glucose transport through the erythrocyte membrane and, in consequence favors HbA1 glycation [32]–[35]. The overestimation of HbA1c levels in patients treated with metformin may have important clinical implications and, therefore, prospective clinical studies, specifically addressed to confirm this issued are needed.

In summary, in type 2 diabetic patients, the GG, defined as the difference between the measured level of A1c and the level that would be predicted from its regression on the fructosamine level, is related to serum creatinine levels and the MCHC. Moreover, there is a higher level of HbA1c relative to fructosamine in patients treated with metformin. This spurious effect of the drug on HbA1c may have significant clinical implications.
